# Qingjie Fuzheng Granule Inhibits EMT and Induces Autophagy in Colorectal Cancer via mTOR Signaling Pathways

**DOI:** 10.1155/2021/9950499

**Published:** 2021-11-30

**Authors:** Xiaoqin Zhu, Yongan Chen, Minghe Lin, Bin Huang, Jiumao Lin

**Affiliations:** ^1^Academy of Integrative Medicine, Fujian Key Laboratory of Integrative Medicine on Geriatrics, Fujian University of Traditional Chinese Medicine, Fuzhou 350122, China; ^2^Key Laboratory of Integrative Medicine (Fujian University of Traditional Chinese Medicine), Fujian Province University, Fuzhou 350122, China; ^3^Department of Oncology, Naval Medical Center of Chinese People's Liberation Army, Shanghai 20000, China

## Abstract

Qingjie Fuzheng granule (QFG) is a traditional Chinese medicinal formula used extensively as an alternative medicine for cancer treatment, including colorectal cancer (CRC). But its pathological mechanism in CRC is unclear. To study antitumor treatment effects and mechanisms of QFG, we established a CRC HCT-116 xenograft mouse model and assessed QFG on EMT and autophagy progression in vivo. The mice were randomly divided into 2 groups (*n* = 10 each group) and treated with intragastric administration of 1 g/kg of QFG or saline 6 days a week for 28 days (4 weeks). Body weight was measured every other day with electronic balance. At the end of the treatment, the tumor weight was measured. Immunohistochemical (IHC) and western blot (WB) assay were used to detect the expression level of E-cadherin, N-cadherin, vimentin, and TWIST1 to evaluate the effect of QFG on tumor cell EMT progression. IHC and WB assay were also used to detect the expression level of beclin-1, LC3-II, and p62 to evaluate the effect of QFG on tumor cell autophagy progression. Furthermore, the expression level of relative proteins in mTOR pathway was detected by WB assay to investigate the mechanism of QFG effect on CRC. We discovered that QFG inhibited the rise of tumor weight while it had no effect on mice body weight, which proved that QFG could inhibit CRC growth progression without significant side effects in vivo. In addition, QFG treatment inhibited EMT and induced autophagy progression in CRC tumor cells, including that QFG upregulated the expression of E-cadherin, beclin-1, and LC3-II, but downregulated the expression of N-cadherin, vimentin, TWIST1, and p62. And, QFG decreased the ratio of p-PI3K/PI3K, p-AKT/AKT, and p-mTOR/mTOR, but increased the ratio of p-AMPK/AMPK. All findings from this research proved that QFG can induce autophagy and inhibit EMT progression in CRC via regulating the mTOR signaling pathway.

## 1. Introduction

Owing to diet structure and lifestyle changes, as well as the growing aging population, colorectal cancer (CRC) has become one of the most prevalent malignancies, accounting for 25% of all cancer-related deaths worldwide [1]. Although diagnosis and treatment technology to CRC continues to progress, there are still 50% patients who could have adverse effects, recurrence, and metastasis [2, 3]. The limitations of CRC therapies mentioned above highlight the need for safer and more selective remedies with fewer adverse effects. Thus, the search for novel therapies has garnered considerable interest worldwide.

Traditional Chinese medicines exhibit relatively fewer adverse effects and have been clinically used as a crucial alternative remedy for CRC therapies. Qingjie Fuzheng granules (QFG), which belong to a traditional Chinese medicine (TCM) formula, are clinically effective for CRC treatments with few adverse effects [4, 5]. In our previous in vitro study, we demonstrated that QFG could inhibit CRC cell proliferation, migration, and invasion and promote CRC cell apoptosis [6–9]. Invasion and metastasis are important characteristics of malignant tumors, and EMT plays a crucial role in invasion and metastasis of cancer cells [10, 11]. EMT makes cancer cells lose the characteristics of epithelial cells and gain the characteristics of mesenchymal cells, so they have stronger invasion and metastasis capabilities [12]. In the study of EMT, it was discovered that the autophagy, as a tightly regulated physiological response activated by metabolic stress and other microenvironmental changes, may participate in the process of EMT, thereby affecting tumor invasion and metastasis [13].

Autophagy is an important mechanism for cells to maintain homeostasis. Through autophagy, it can degrade senescent proteins and organelles and promote or inhibit tumor development. Therefore, it is a “double-edged sword” in cancer [14]. At different stages of cancer development, autophagy may participate in the process of EMT through different molecular mechanisms and then affect tumor invasion and metastasis. In the early stages of cancer metastasis, autophagy suppresses cancer initiation through eliminating inflammation and tissue damage to reverse EMT in cancer cells and to inhibit early metastasis [15]. Later on, as a tumor grows, autophagy provides the energy to promote the invasion of cancer cells by inducing the EMT in some types of tumors, such as hepatocellular carcinoma and pancreatic cancer [16–18]. These indicate that there is an intricate relationship between the autophagy and EMT in cancer. At present, the regulatory mechanism of autophagy to the EMT in cancer is not completely clear. Some studies have found that autophagy and EMT are usually regulated by PI3K/AKT/mTOR, beclin-1, p53, and JAK/STAT signaling pathways [19]. The PI3K/AKT/mTOR signaling pathway plays an important role in regulating autophagy and EMT.

In a word, autophagy and EMT play an important role in the occurrence and development of cancers. Therefore, we should try our best to discover some drugs that can regulate autophagy and inhibit EMT, which must be beneficial for cancer treatment. There are no studies which have been reported on the effect and mechanism of QFG on CRC cell EMT and autophagy which are also the key regulatory factors in the progression of CRC. Hence, in the present study, we aim to explore the effect of QFG on EMT and autophagy in the CRC process. We also elucidate the underlying molecular mechanisms.

## 2. Materials and Methods

### 2.1. Preparation of QFG

QFG were obtained and prepared as previously described [20]. In brief, QFG powder were stored at 4°C. Before using, QFG powder was dissolved by saline (cat. no. R22173; Yuanye Biology; Shanghai, China).

### 2.2. Cell Culture

Human colon carcinoma HCT-116 cell line was purchased from the American Type Culture Collection. The cell was cultured in RPMI-1640 (cat. no. C11875500BT; Life Technologies Corp. Grand Island, USA) complete medium which contains 10% FBS and 1% penicillin/streptomycin and grown in a humidified atmosphere with the condition of 5% CO_2_, 37°C.

### 2.3. Animals and In Vivo Mice Xenograft Study

20 six-week-old male BALB/c nude mice (Shanghai SLAC Laboratory Animal Co., Ltd. Shanghai, China) whose weight is about 20–22 g were raised in an SPF-controlled environment by keeping a 12 h light/dark cycle under ad libitum access to food and water. 2 × 10^6^ of HCT-116 cells were mixed with equal volume of PBS and Matrigel (cat. no. 354248; Corning, USA) and then injected the cells in the right flank area of each mouse subcutaneously. When the size of the tumor was approximately at 100–300 mm^2^, mice were divided into the control group and QFG-treated group (*n* = 10 for each group) randomly. Control group was given intragastric administration of saline, and QFG-treated group was treated with 1 g/kg of QFG, continuously six days a week for 28 days. Bodyweight and tumor size were measured every other day with electronic balance and caliper. The formula for calculating tumor volume is as follows: major diameter (*L*) × (minor diameter)^2^ (*W*^2^) × *π*/6. After 28 days of treatment, the mice were sacrificed with diethyl ether, and the tumor tissue was harvested and weighed. Six tumor tissues per group were selected randomly and fixed with 4% paraformaldehyde for immunochemistry (IHC) assay. Four tumor tissues per group were selected randomly for following western blot assay. All experiments were repeated at least 3 times. The experimental procedures and the care of animals were performed strictly according to international ethical guidelines and the Guidance Suggestions for the Care and Use of Laboratory Animals issued by the Ministry of Science and Technology of the People's Republic of China. This experiment was approved by the Institutional Animal Care and Use Committee of Fujian University of Traditional Chinese Medicine (no. 2019009).

### 2.4. Immunohistochemical (IHC) Assays

IHC assays were performed as described previously [5]. In brief, tumor sections (5 *μ*m thick) were, respectively, treated with antibody of LC3-II (cat. no. #3868), (1 : 500, Cell Signaling Technology, Beverly, MA, USA), beclin-1 (cat. no. 11306-1-AP), p62 (cat. no. 18420-1-AP), E-cadherin (20874-1-AP), N-cadherin (22018-1-AP), vimentin (60330-1-Ig), and TWIST1 (25465-1-AP) (1 : 200; Proteintech, USA) overnight at 4°C. Then, sections were incubated with a corresponding secondary antibody for 30 min and treated with the ABC reagent for 30 min and then treated with 3,3′-diaminobenzidine for 10 min. Finally, the detected indexes were observed and documented (400×) by a microscope (Leica, Solms, Germany). The quantification of IHC assays was calculated through the rate of positive cell number to total cell number at 5 fields which were selected in each slide randomly.

### 2.5. Western Blot Analysis

Four tumors were selected randomly from the control or QFG group, homogenized in nondenaturing lysis buffer using homogenizer, and centrifuged at 15,000 × *g* for 15 min followed by determination of protein concentration in supernatants. Equal protein per lysate was resolved on tris-glycine gel and transferred onto the PVDF membrane. 5% nonfat dry milk was used to block the PVDF membranes for 1 h, and then primary antibodies beclin-1 (cat. no. 11306-1-AP), p62 (cat. no. 18420-1-AP), E-cadherin (cat. no. 20874-1-AP), N-cadherin (cat. no. 22018-1-AP), vimentin (cat. no. 60330-1-AP), TWIST1 (cat. no. 25465-1-AP), PI3K (cat. no. 13329-1-AP), AKT (cat. no. 10176-2-AP), AMPK (cat. no. 10929-2-AP), mTOR (cat. no. 20657-1-AP) (1 : 1000, Proteintech, USA), p-PI3K (cat. no. ab-110021) and p-AKT (cat. no. ab-15285) (1 : 1000, Abcam, CA, USA), LC3-II (cat. no. #3868), p-AMPK (cat. no. #50081), p-mTOR (cat. no. #5536), and *β*-actin (cat. no. #4967) (1 : 1000, Cell Signaling Technology) were added at 4°C overnight. On the second day, the appropriate HRP-conjugated secondary antibodies—goat anti-mouse IgG secondary antibody (cat. no. #L3032) and goat anti-rabbit IgG secondary (cat. no. #L3012) (1 : 5000, Signalway Antibody, PA, USA)—were incubated with these membranes at room temperature for 1 hour. *β*-actin was used as the internal reference. The immunoreactive protein signals were detected by SuperSignal West Pico Chemiluminescent Substrate. The densitometry of the gel bands was analyzed using Bio-Image Analysis System (Bio-Red, Hercules, CA, USA).

### 2.6. Statistical Analysis

The data analysis was performed with SPSS software (version 21.0). The relationship between two groups was analyzed using *t*-test. Mean ± standard deviation indicated the statistical data. All experiments were conducted three times. Statistically significant result was labeled by *P* < 0.05.

## 3. Results

### 3.1. QFG Inhibits HCT-116 Tumor Xenograft Growth in Nude Mice

**Figure 1:**
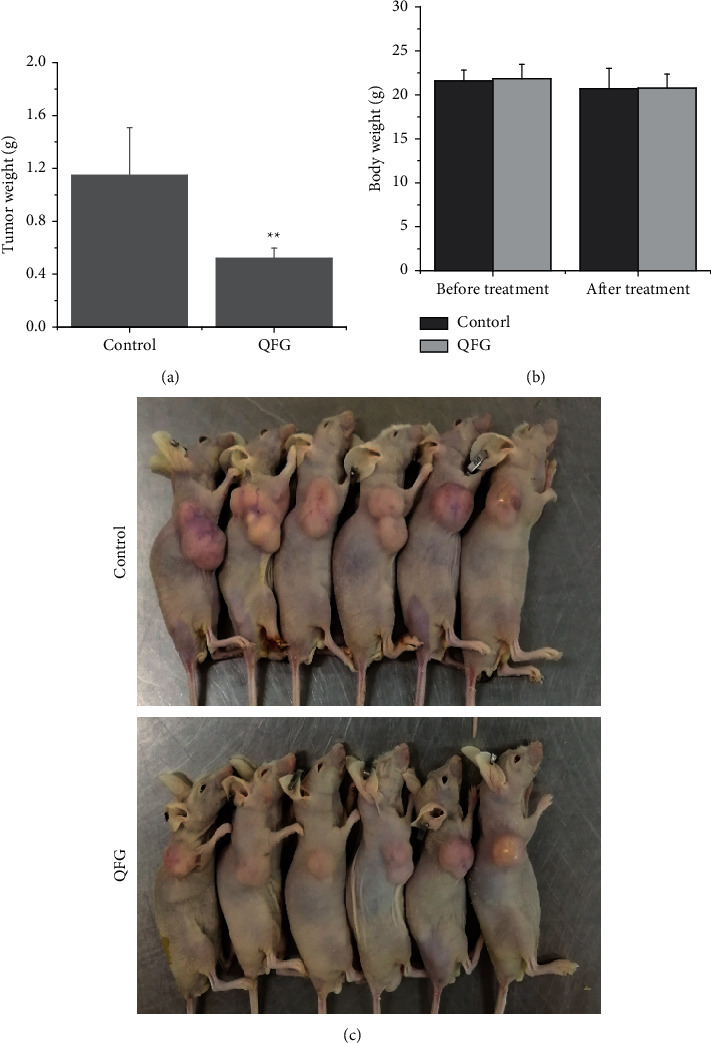
Effect of QFG on HCT-116 xenograft tumor growth and body gain in mice. (a) Tumor weight (g) of per nude mouse was measured at the end of the study. (b) Body weight (g) of per nude mouse was measured before and after treatment. (c) The pathological findings of solid tumor from the back of the mice after HCT-116 cell implantation and treatment for 28 days. ^*∗∗*^*P* < 0.01, QFG treatment group vs. control group.

To study the effect of QFG on tumor growth, we detected tumor weight in all CRC xenograft mice and then compared this data in QFG-treated group with control group. As exhibited in Figure 1(a), tumor weight in the QFG-treated group on the last day was 0.64 ± 0.49 g, whereas that in the control group was 1.15 ± 0.36 g (*P* < 0.01). Conversely, the body weight had no change after treatment compared with the before-treatment group both in the QFG-treatment and control group (Figure 1(b)). In addition, in Figure 1(c), the picture of solid tumor intuitive verified those results in Figures 1(a) and 1(b). In all, it is indicated that QFG is effective in inhibiting colorectal tumor growth, without significant toxicity.

### 3.2. QFG Inhibits EMT Progression in CRC Tumor Cells by Regulating the Expression of E-Cadherin, N-Cadherin, Vimentin, and TWIST1

**Figure 2:**
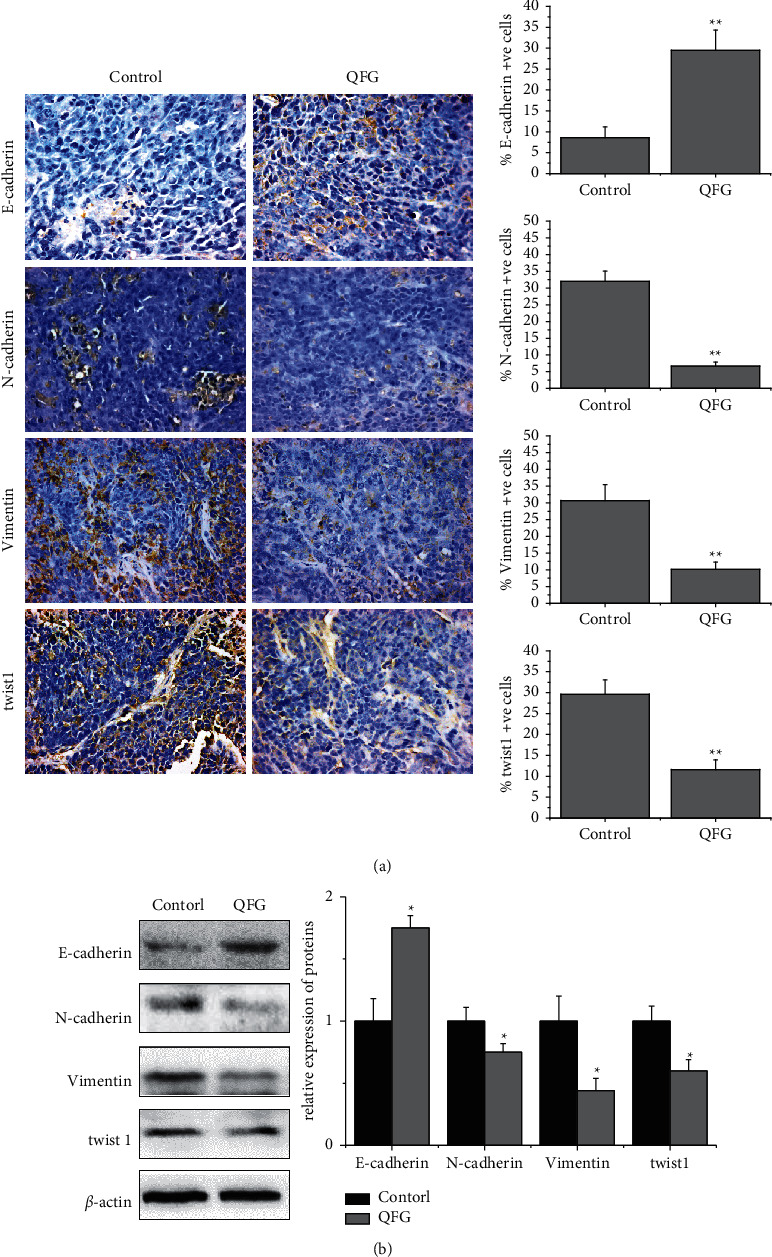
The expression of E-cadherin, N-cadherin, vimentin, and TWIST1 in tumor cells of CRC xenograft mice in each group. (a) Immunohistochemistry stain (400×). Quantification of IHC assay was represented as percentage of positively stained cells ($\bar{X}$ ± *S*, *n* = 6). (b) Protein expression was detected by western blot ($\bar{X}$ ± *S*, *n* = 4). *β*-Actin was used as the internal control. ^*∗*^*P* < 0.05 and ^*∗∗*^*P* < 0.01, QFG treatment group vs. control group.

During the epithelial-to-mesenchymal transition (EMT) process, some vital protein expressions are promoted, which are characterized by the absence or downregulation of epithelial cell differentiation characteristics and the acquisition of mesenchymal markers [16]. E-cadherin is the epithelial marker, while N-cadherin and vimentin are the important mesenchymal markers [21]. In this study, the impact of QFG on CRC tumor cell EMT progression was determined using IHC and western blot assays to detect the expressions of these markers. As shown in Figure 2(a), compared with the control group, QFG treatment obviously upregulated the expression level of E-cadherin, while downregulated the expression level of N-cadherin and vimentin (*P* < 0.01). In addition, TWIST1 is the key transcription factor that controls initiation of EMT [22]. So, we also detected the expressions of TWIST1 by IHC and western blot assays. As shown in Figure 2(a), compared with the control group, QFG treatment obvious reduced the expression level of TWIST1 in CRC tumors (*P* < 0.01). The result of western blot assay was consistent with IHC assay (Figure 2(b), *P* < 0.05), which proved that QFG can inhibit the progression of EMT in CRC tumor cells.

### 3.3. QFG Induce Autophagy in CRC Tumor Cells by Regulating LC3-II, Beclin-1, and p62

**Figure 3:**
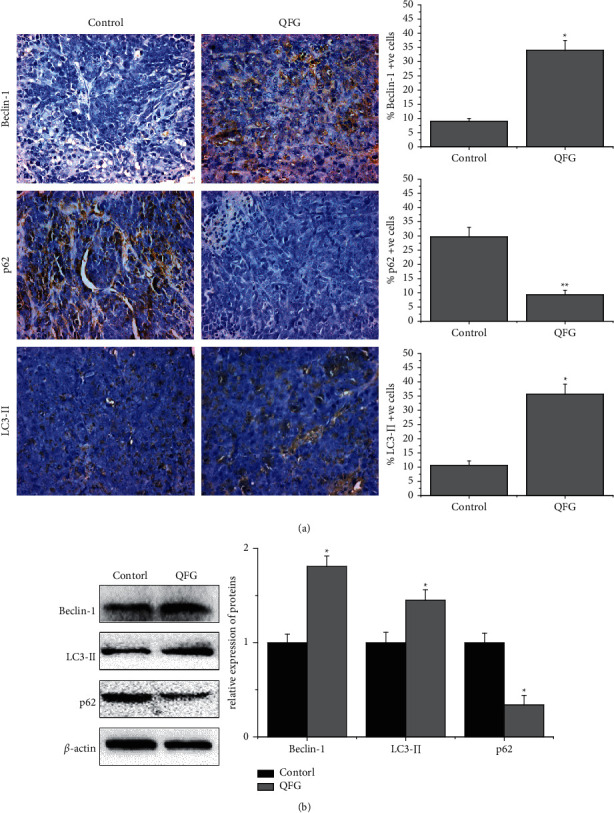
The expression of LC3-II, beclin-1, and p62 in tumor cells of CRC xenograft mice in each group. (a) Immunohistochemistry stain (400×). Quantification of IHC assays were represented as percentage of positively stained cells ($\bar{X}$ ± *S*, *n* = 6). (b) Protein expression detected by western blot ($\bar{X}$ ± *S*, *n* = 4). *β*-Actin was used as the internal control. ^*∗*^*P* < 0.05 and ^*∗∗*^*P* < 0.01, QFG treatment group vs. control group.

Accumulated evidence has suggested that there is an association between autophagy and EMT [19]. So, we further detected the effect of QFG on autophagy in CRC tumor cells. Autophagy in tumors was examined by detecting the expression of LC3-II which is widely used as an autophagosome marker [23, 24]. As shown in Figure 3(a), in the control group, the LC3-II-positive cells' percentage in tumor tissues was 10.67 ± 1.53%, while that in the QFG-treated group was 35.67 ± 3.51% (*P* < 0.05), which demonstrated that QFG has induced the autophagy effect in vivo. To further verify this result, we detected the effect of QFG on the expression of beclin-1 and p62 which are key regulators of autophagy. As shown in Figure 3(a), compared with the control group, QFG upregulated beclin-1 and p62 expressions (*P* < 0.05 and *P* < 0.01, respectively). The result of western blot assay was consistent with IHC assay (Figure 3(b)), which verified that QFG can induce autophagy in CRC tumor cells.

### 3.4. QFG Suppresses mTOR Signaling Pathways in CRC Xenograft Mice

**Figure 4:**
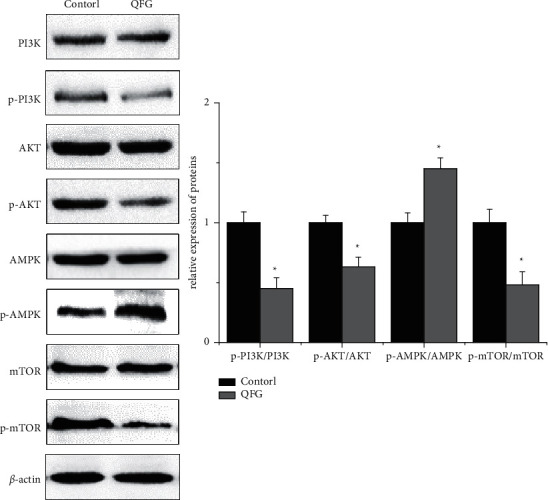
The protein expression of PI3K, p-PI3K, AKT, p-AKT, AMPK, p-AMKP, mTOR, and p-mTOR in tumor cells of CRC xenograft mice was detected by western blot in each group ($\bar{X}$ ± *S*, *n* = 4). *β*-Actin was used as the internal control. ^*∗*^*P* < 0.05, QFG treatment group vs. control group.

To further investigate the potential mechanisms of this effect of QFG on CRC tumor, we examined the expression of major regulatory factors involved in the mTOR signal pathway which major regulated EMT and autophagy progression. Western Blot was used to detect the expression level of PI3K, p-PI3K, AKT, p-AKT, AMPK, p-AMPK, mTOR, and p-mTOR, as shown in Figure 4; after QFG treatment, the ratios of p-PI3K/PI3K, p-AKT/AKT, and p-mTOR/mTOR were significantly decreased compared with the control group, while the ratio of p-AMPK/AMPK was increased compared with the control group (*P* < 0.05, respectively). All these results indicated that QFG can inhibit activation of the mTOR signaling pathway to inhibit the progression of EMT and autophagy in CRC tumor.

## 4. Discussion

CRC is one of the most common digestive malignant tumors, which seriously endangers human survival and health. It is very important to find effective anti-CRC drugs and elucidate their underlying molecular mechanisms. QFG is a four-herb TCM formula, consisting of *Hedyotis diffusa* Willd, *Scutellaria barbata* D. Don, malt, and astragalus, which are usually used as an anti-CRC drug in clinical applications [4, 5]. In the past few years, some researches have proved that *Hedyotis diffusa* Willd and *Scutellaria barbata* D. Don are capable of promoting apoptosis and inhibiting growth in varies types of cancer cells, including CRC [25, 26]. In addition, previous *in vitro* and *in vivo* studies had proved that QFG can inhibit proliferation, migration, and invasion and induce apoptosis in cancer cells [6–9]. However, no studies have not been reported on the effect and mechanism of QFG on CRC cell autophagy and EMT which are also the key regulatory factors in the progression of CRC. So, the aim of this research is to investigate the effect and mechanism of QFG on autophagy and EMT in CRC progression.

In this study, a CRC mouse xenograft model was used to evaluate the treatment effect and molecular mechanisms of QFG on CRC progression and discovered that QFG inhibited the increase of tumor weight at the same time and had no effect on body weight in CRC mice, proving that QFG could inhibit CRC progression *in vivo*. Accumulating research has suggested that EMT, which is a critical biological behavior involved in proper embryonic development, has a close correlation with the migratory and invasive abilities of cancer cells [15]. During the EMT process, some vital proteins expressions are promoted, which is characterized by the absence or downregulation of epithelial cell differentiation characteristics and the acquisition of mesenchymal markers [27]. So, the expression level of some molecular markers could reveal the extent of EMT; among these molecular markers, E-cadherin is the epithelial marker, while N-cadherin and vimentin are the important mesenchymal markers [19]. In the progression of malignancy, the downregulation of E-cadherin expression, the transformation of E-cadherin to N-Cadherin on the cell membrane surface, and the conversion of cytokeratin cytoskeleton into vimentin-based cytoskeleton (increased expression of vimentin) indicate that the adhesion ability of tumor cells is decreased, and the ability of movement, invasion, and metastasis is enhanced. In this study, we found QFG could increase the expression level of E-cadherin but decrease the expression of N-cadherin and vimentin, which indicated that QFG can inhibit the EMT progression in CRC. In addition, TWIST1 is a helix-loop-helix transcription factor, which can suppress the expression of E-cadherin, reduce cellular adhesion, and increase motility [28]. In this study, we also found QFG inhibited the expression level of TWIST1, which indicated QGFs can inhibit EMT progression via regulating TWIST1 in CRC.

Emerging observations indicate that there is an intricate relationship between autophagy and metastasis in cancer. Autophagy is a lysosomal catabolic pathway that degrades protein aggregates and excess or damaged organelles before canceration to maintain normal cellular structure and metabolic stability [29]. Autophagy suppresses tissue damage, inflammation, and genome instability that can promote cancer initiation via its quality control function; therefore, activating autophagy may be conducive to change the disorder state of proteins in the body, keep the internal environment stable, protect cells from DNA mutation and canceration, and inhibit the formation of tumors [30]. During autophagy progression, LC3 plays a significant role in the formation of autophagosomes through a mechanism related to the autophagosome membrane [31]. LC3 protein is present in two forms: LC3-I and LC3-II. LC3-I localizes in the cytoplasm, whereas LC3-II is membrane bound and enriched in both inside and outside autophagosomes [32]. In addition, LC3-II, used as a marker of autophagy, might regulate the formation of autophagosomes and control the number of autophagosomes [30]. In this study, we demonstrated that QFG can promote the expression of LC3-II in CRC tumor cells, which proved that QFG can induce the autophagy progression in CRC. And, our result also indicated QFG increased the expression of beclin-1 which is the upstream regulator of LC3 and further verified this conclusion. Sequestosome1 (p62/SQSTM 1) is a multidomain protein that binds to ubiquitinated protein and then interacts with LC3 through the LIR domain to facilitate the clearance of ubiquitinated and autophagic cargos [32, 33]. Under normal physiological conditions, p62 is constantly degraded by the autophagy-lysosome system [34]. Autophagy inhibition and autophagy deficiency can cause p62 accumulation [35]. In this study, we demonstrated QFG decreased the expression of p62 which indicated that QFG induces autophagy in CRC via regulating p62.

In the process of tumor cell invasion and metastasis, autophagy and EMT are closely linked in a complex relationship and play an important role [16]. Autophagy may play a “dual role” in regulating the EMT process through different molecular mechanisms. On the one hand, autophagy promotes the occurrence and development of EMT [17, 18]. On the other hand, autophagy can be used as a tumor suppressor signal by selectively regulating key mediators such as p62, TWIST1, and beclin-1 to counteract the activation of EMT [36, 37]. Beclin-1-induced autophagy by forming the PI3K complex and inhibited EMT by downregulating vimentin and TWIST1 expression and increasing E-cadherin expression [38]. In this study, we found that QFG upregulated the expression of E-cadherin and beclin-1 while downregulated the expression of N-cadherin, vimentin, and TWIST1 in CRC cells. The results may indicate that QFG-induced autophagy might inhibit the EMT in CRC cells.

The dual effect of autophagy on EMT is regulated by multiple signaling pathways, such as PI3K/AKT/mTOR, AMPK/mTOR, MAPK/ERK1/2, and HGF/MET pathways [15, 39]. Among these pathways, PI3K/AKT/mTOR and AMPK/mTOR, which collectively called as mTOR signaling pathway, plays a vital role in the process of autophagy and EMT. In this pathway, phosphorylated PI3K promotes the phosphorylation of AKT, and then phosphorylated AKT promotes the activation of mTOR which inhibits the development of autophagy [15, 39, 40]. At the same time, phosphorylated AMPK can inhibit the activation of the mTOR signaling pathway, and it positively regulates autophagy through increasing AMPK phosphorylation and decreasing mTOR phosphorylation [41]. Further research suggests that induced autophagy can inhibit EMT, and defects in autophagy will promote EMT. Autophagy deficiency stabilizes TWIST1 protein through the accumulation of p62/SQSTM 1. SQSTM 1 binds to TWIST1 to inhibit the degradation of TWIST1 by autophagosomes and proteasomes and promote the occurrence of EMT [42]. Moreover, it was reported that the PI3K/Akt pathway positively regulates Wnt/*β*-catenin through phosphorylating GSK-3*β* at Ser9, which is capable of increasing the expression of intracellular *β*-catenin that combines with E-cadherin to promote EMT [43]. And, the present research demonstrated that QFG downregulated the ratio of p-PI3K/PI3K, p-AKT/AKT, and p-mTOR/mTOR, while upregulated the ratio of p-AMPK/AMPK, which showed that QFG may induce autophagy in CRC via the mTOR signaling pathway and can inhibit the EMT by inducing autophagy and decreasing the level of TWIST1 and increasing the level of beclin-1.

## 5. Conclusion

In conclusion, this study demonstrated that QFG could be valuable for its inhibitory effects against CRC. The results showed that QFG induced autophagy in CRC by regulating the mTOR pathway and inhibited the progression of EMT by inducing autophagy and decreasing the level of twist1 and increasing the level of beclin-1. It indicated that QFG is a potential new therapeutic drug for CRC.

## Data Availability

The datasets used and analyzed during the current study are available from the corresponding author on reasonable request.
